# And Now There Are Five: A New Player in Intracellular Trafficking Pathways

**DOI:** 10.1371/journal.pbio.1001173

**Published:** 2011-10-11

**Authors:** Stephanie Huang

**Affiliations:** Freelance Science Writer, Sunnyvale, California, United States of America

**Figure pbio-1001173-g001:**
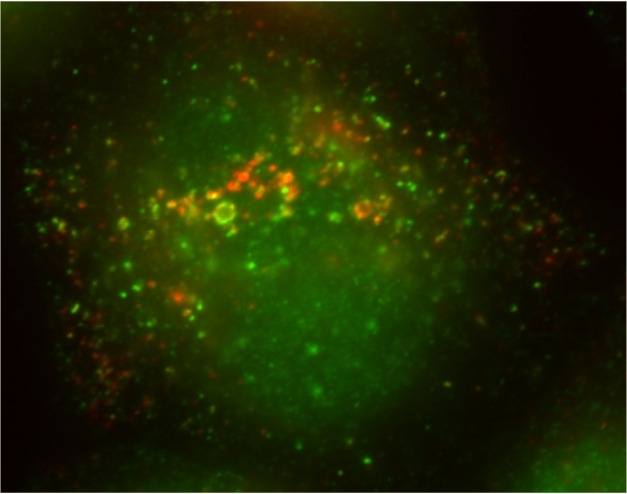
The new adaptor protein (AP) complex, AP-5, labeled in green, is partially associated with the organelles labeled in red, which are late endosomes and lysosomes.

[Fig pbio-1001173-g001]Peering at a cell through a microscope reveals a hotbed of seemingly random activity, as proteins zip here and there. But the business of shuttling proteins to the correct destination is in fact a highly regulated, high-stakes affair. Disruption of these trafficking pathways can affect a cell's interaction with the surrounding cells and external environment. Neuronal cells, for example, rely on trafficking pathways to properly send and receive chemical signals. Mutations in genes involved in trafficking are implicated in a number of neurodegenerative disorders—including Alzheimer's and Huntington's disease, amyotrophic lateral sclerosis (ALS), and hereditary spastic paraplegia—underscoring the importance of understanding this basic cellular process.

Specialized structures in the cell—including organelles such as endosomes, lysosomes, and the trans-Golgi network (TGN)—operate much like FedEx shipping centers, sorting and routing protein cargo to make sure they reach their final destination. Until now, researchers had identified four molecular FedEx drivers, called adaptor protein (AP) complexes, that work the different routes connecting endosomes, lysosomes, and the TGN. In this issue of *PLoS Biology*, Jennifer Hirst, Joel Dacks, Margaret Robinson, and colleagues report the identification of a fifth AP complex, helping to paint a more complete picture of protein trafficking in the cell.

On their way into or out of the cell, cargo proteins may pass through multiple organelles, transported inside small membrane “bubbles” called vesicles. Generally speaking, outbound cargo will pass through the TGN to the tubular endosome, and then out to the plasma membrane. Inbound cargo will enter the cell at the plasma membrane, pass through the early endosome, and may stop at the late or tubular endosomes, before reaching the lysosomes or TGN. Each AP complex is assigned to a distinct route—for example, AP-2 handles the route from the plasma membrane to the early endosome, and AP-1 handles the route between the tubular endosomes and the TGN. Along their respective routes, the AP complexes recognize and bind to specific cargo proteins and gather them into a vesicle for transport.

AP complexes are composed of four protein subunits—two large proteins, one medium protein, and one small protein. While studying a poorly understood protein called C14orf108, Hirst et al. discovered that the protein is very similar in size, sequence, and predicted shape to the medium-sized proteins of AP complexes. The researchers also found that C14orf108 interacts with a novel protein called DKFZp761E198, which is similar to the large proteins found in AP complexes.

Suspecting that they had identified a new AP complex, Hirst et al. devised further experiments to test whether these two proteins might be involved in trafficking. C14orf108 appears to reside in the late endosomes and lysosomes, consistent with a role in trafficking. Making use of cargo proteins that represent well-studied trafficking routes, the researchers determined that the route between the TGN and endosomes was affected when cells were depleted of either C14orf108 or DKFZp761E198. Detailed imaging under the electron microscope revealed that these same cells contained swollen endosome-related structures with extended tubules—as if bubble-like vesicles had partially formed but could not separate. The team concluded that they had in fact identified a new AP complex—dubbed AP-5—that may pick up cargo from late endosomes or lysosomes.

Relatively little is understood about trafficking routes originating from late endosomes—with the identification of this new complex, researchers in the field now have a new lead for investigating these routes.

Because AP complexes usually contain four protein subunits, the researchers searched for the remaining two proteins that would make up the rest of the AP-5 complex. They noted that another research group, Mikołaj Słabicki et al., had recently published findings in *PLoS Biology* indicating that two proteins called SPG48 and C20orf29 interact with C14orf108 and DKFZp761E198. Intriguingly, SPG48 and C20orf29 appear to fit the criteria for the remaining two protein subunits of the new AP-5 complex—they are the right size and their predicted shape resembles the corresponding proteins in other AP complexes. Depleting cells of either protein disrupted the trafficking route between the TGN and endosomes, the same route affected by depletion of C14orf108 and DKFZp761E198. Hirst et al. independently confirmed that the four proteins interact with each other, leading them to conclude that they make up the AP-5 complex.

By comparing the genomic sequences of 29 eukaryotic species, the team determined that this complex emerged fairly early in the evolution of eukaryotic cells. The analysis also suggested that AP-5 had disappeared in many species, including the commonly studied baker's yeast, which could help explain why it had not been identified until now. Based on their genomic analyses, the team hypothesized that the AP-3 complex was the earliest complex to evolve, followed by AP-5, AP-4, and finally AP-1 and AP-2.

The study from Słabicki et al. identified SPG48 as a new gene associated with hereditary spastic paraplegia, a group of genetic disorders characterized by progressive weakness and stiffness of the legs. Although a number of genes have been identified to underlie forms of hereditary spastic paraplegia, researchers have yet to develop treatments to prevent, slow, or reverse the disease. Identification of the AP-5 complex not only reveals a new player involved in sparsely studied trafficking pathways, but also offers researchers a potential new lead in understanding the genetic basis of hereditary spastic paraplegia, as well as other neurodegenerative diseases that arise from trafficking defects.

**Hirst J, Barlow L, Francisco GC, Sahlender DA, Seaman MNJ, et al. (2011) The Fifth Adaptor Protein Complex. doi:10.1371/journal.pbio.1001170**

